# Cfr-Mediated Linezolid-Resistance among Methicillin-Resistant Coagulase-Negative Staphylococci from Infections of Humans

**DOI:** 10.1371/journal.pone.0057096

**Published:** 2013-02-20

**Authors:** Lanqing Cui, Yang Wang, Yun Li, Tao He, Stefan Schwarz, Yujing Ding, Jianzhong Shen, Yuan Lv

**Affiliations:** 1 Institute of Clinical Pharmacology, Peking University First Hospital, Peking University, Beijing, People's Republic of China; 2 College of Veterinary Medicine, China Agricultural University, Beijing, People's Republic of China; 3 Institute of Farm Animal Genetics, Friedrich-Loeffler-Institut (FLI), Neustadt-Mariensee, Germany; Institut National de la Recherche Agronomique, France

## Abstract

Four methicillin-resistant coagulase-negative staphylococci (MRCoNS), one *Staphylococcus haemolyticus* and three *Staphylococcus cohnii*, from infections of humans collected via the Ministry of Health National Antimicrobial Resistance Surveillance Net (Mohnarin) program in China were identified as linezolid-resistant. These four isolates were negative for the 23S rRNA mutations, but positive for the gene *cfr*. Mutations in the gene for the ribosomal protein L3, which resulted in the amino acid exchanges Gly152Asp and Tyr158Phe, were identified in *S. haemolyticus* 09D279 and *S. cohnii* NDM113, respectively. In each isolate, the *cfr* gene was located on a plasmid of *ca.* 35.4 kb, as shown by S1 nuclease pulsed-field gel electrophoresis and Southern blotting experiments. This plasmid was indistinguishable from the previously described plasmid pSS-02 by its size, restriction pattern, and a sequenced 14-kb *cfr*-carrying segment. Plasmid pSS-02 was originally identified in staphylococci isolated from pigs. This is the first time that a *cfr*-carrying plasmid has been detected in MRCoNS obtained from intensive care patients in China. Based on the similarities to the *cfr*-carrying plasmid pSS-02 from porcine coagulase-negative staphylococci, a transmission of this *cfr*-carrying plasmid between staphylococci from pigs and humans appears to be likely.

## Introduction

Linezolid is an important antimicrobial agent for the therapy of infections caused by gram-positive pathogens, especially methicillin-resistant S*taphylococcus aureus* and vancomycin-resistant enterococci. Linezolid is available in almost 70 countries, and has been used to treat approximately four million patients since it has been approved for clinical use in the U.S.A. in 2000 [1]. Resistance to linezolid was first reported in a methicillin-resistant *Staphylococcus aureus* (MRSA) clinical isolate in 2001 [2]. Since then, the occurrence of linezolid-resistant staphylococci has been increasingly reported in Europe (e.g. in Ireland and Spain) and in the United States [3].

Resistance to oxazolininones can be based on mutations in the central loop of the 23SrRNA gene with the substitution G2576T occurring most frequently; substitutions for T2500A, T2504A and G2215A have also been found in staphylococcal isolates from clinical infections, while G2444T, G2447T, A2503G and T2504C have so far only been found among laboratory-derived *Staphylococcus* strains [3], [4]. Moreover, elevated linezolid MICs can also be associated with mutations in the genes for the ribosomal proteins L3 and L4, some regions of which interact closely with the linezolid binding site in the peptidyltransferase center [5], [6]. More recently, the transferable multiresistance gene *cfr*, originally identified in a bovine *Staphylococcus sciuri* isolate, was found to code for a RNA methyltransferase which modifies the adenine residue at position 2503 in the 23S rRNA and thereby confers resistance not only to oxazolidinones, but also to phenicols, lincosamides, pleuromutilins, and streptogramin A antibiotics (otherwise known as the PhLOPS_A_ phenotype) [7]. To date, the *cfr* gene has been found in staphylococci from clinical cases isolated from Colombia, the United States, Italy, Spain, Ireland and Mexico [8], [9], [10], [11], [12], [13], [14], [15], [16].

In China, linezolid was first approved for use in clinical practice in 2007. Since then, there has been only one report of linezolid resistant methicillin-resistant coagulase-negative staphylococci, and this occurred in an intensive care unit (ICU) of a Chinese hospital [17]. In the respective study, the *cfr* gene was detected by PCR, but neither a plasmid location of the *cfr* gene could be confirmed nor the genetic environment of the *cfr* gene be determined. The present study was conducted to investigate four clinical linezolid-resistant *Staphylococcus* spp. isolates collected from the Ministry of Health National Antimicrobial Resistance Surveillance Net (Mohnarin) program in China for the presence and the location of the *cfr* gene, but also linezolid resistance-mediating mutations which may be present in the same isolates.

## Materials and Methods

### Bacterial isolates

Four linezolid-resistant coagulase-negative *Staphylococcus* isolates, including one *Staphylococcus haemolyticus* (09D279) and three *Staphylococcus cohnii* isolates (09D253, 09D363 and NDM113), were identified among 713 clinical staphylococcal isolates by growth on brain heart infusion (BHI) agar containing 6 µg/ml linezolid. All isolates were collected between 2009 and 2010 from 19 hospitals that participated in the Mohnarin program. These hospitals are located in 17 cities that are widely distributed across China. The four isolates (09D279, 09D253, 09D363 and NDM113) were obtained from blood cultures collected from four ICU patients, all of which were over 60 years of age. Further information about these patients, the underlying disease and possible antimicrobial pretreatment were unfortunately not available. The *S. haemolyticus* 09D279 collected in January 2010 and the *S. cohnii* isolates 09D253 collected in December 2009 and 09D363 collected in March 2010 were obtained from individual patients in hospital A in Shenyang, Liaoning province, whilst *S. cohnii* NDM113 collected in October 2010 was isolated from a patient in hospital B in Beijing. Two linezolid-susceptible isolates *S. haemolyticus* 09D044 and *S. cohnii* 09D071 (both with MIC values of 0.5 µg/ml) from hospital A were included as internal negative controls for the sequencing approaches. *S. aureus* RN4220 served as the recipient strain for transformation experiments.

### Antimicrobial susceptibility testing

The antimicrobial susceptibilities of the four clinical isolates and the *S. aureus* RN4220 reference strain and its transformants were determined using the agar dilution method according to the recommendations given in document M100-S22 [18] of the Clinical and Laboratory Standards Institute (CLSI). *S. aureus* ATCC®29213 served as a quality strain for susceptibility testing.

### DNA extraction and detection of linezolid resistance-mediating mutations and the multiresistance gene *cfr*

Whole-cell DNA from the *Staphylococcus* isolates was isolated using a commercial kit (TianGen, Beijing, China) according to the manufacturer's instructions. Plasmid DNA was extracted withthe QIAGEN plasmid extraction Midi Kit (Qiagen, Hilden, Germany) using a previously described modification [19]. To detect potential mutations involved in linezolid resistance, the genes *rplC* and *rplD*, coding for ribosomal proteins L3 and L4, respectively, and partial sequences of 23S rDNA were amplified and sequenced using previously described primers [20], [21]. The detection of the *cfr* gene also followed a previously described PCR assay [19].

### Molecular typing and analysis of *cfr*-carrying plasmids

To determine their genetic relatedness, the three linezolid-resistant *S. cohnii* isolates were subjected to pulsed-field gel electrophoresis (PFGE) according to a protocol described previously [19]. To analyze the location of the *cfr* gene, S1 nuclease-PFGE and Southern blot analysis were performed. Briefly, whole-cell DNA of the four linezolid-resistant isolates embedded in agarose gel plugs was treated with S1 nuclease (TaKaRa, Dalian, China) and separated by PFGE alongside a standard low PFG marker (NEB, UK). Subsequently, Southern blot analysis was performed using a DNA probe specific for the *cfr* gene, which was non-radioactively labeled with a DIG High Prime DNA labeling and detection kit (Roche Diagnostics, Mannheim, Germany) as described previously [19]. In addition, the purified plasmids extracted from each of the four original strains were transformed into the *S. aureus* recipient strain RN4220 by electrotransformation [22]. Transformants were selected by incubation for 24 h on BHI agar supplemented with 10 µg/ml florfenicol. The transformants were screened for the presence of plasmids by S1-PFGE and their resistance phenotypes were determined. The sizes of the *cfr*-carrying plasmids extracted from the transformants were estimated by calculation of the sums of the different fragment sizes obtained after BglII digestion.

### DNA sequencing

The partial nucleotide sequences of the *cfr*-carrying plasmids extracted from the transformants were determined by primer walking (Invitrogen, Beijing, China), or a modified random primer sequencing walking strategy [19]. The sequences obtained were annotated using the VectorNTI program (Invitrogen, Carlsbad, CA), and the predicated coding sequences were identified using GLIMMER software. The DNA sequences and deduced amino acid sequences were compared to those deposited in GenBank using the BLAST program (http://blast.ncbi.nlm.nih.gov/Blast).

### Nucleotide Sequence Accession Number

The nucleotide sequences of a 13,976-bp fragment of cfr-carrying plasmid have been deposited in GenBank with the accession number JX827253.

## Results and Discussion

### Identification of linezolid resistance-mediating mutations and the gene *cfr*

All four linezolid-resistant staphylococcal isolates were PCR-positive for the *cfr* gene, and the sequences of the *cfr* amplicons obtained from these isolates were identical to one another and to the corresponding *cfr* sequence of plasmids pSCFS1 (GenBank acccession number AJ579365) in *S. sciuri*, pSS-01 (JQ041372) in *S. cohnii*, pSS-02 (JF834910) in *Staphylococcus saprophyticus*, pSCFS6 (AM408573) in *Staphylococus warneri* and pSCFS7 (FR675942) in ST8-MRSA-IVa/USA300, and also shared 99.9% sequence identity with *cfr* from pSCFS3 in *S. aureus* (AM086211). In addition, all four *cfr*-carrying staphylococcal isolates showed wild-type sequences of the 23S rDNA and the gene *rplD* for the L4 protein through PCR. Alterations were detected in the gene *rplC*, which resulted in amino acid substitutions Gly152Asp and Tyr158Phe in the L3 proteins of *S. haemolyticus* 09D279 and *S. cohnii* NDM113, respectively. The Gly152Asp substitution in the L3 protein has been implicated indirectly in reducing the affinity of oxazolidinones for its target through perturbation of bases 2505 and 2506 in the coding sequence [23]. In addition, in this study, the Tyr158Phe substitution in the L3 protein of *S. cohnii* NDM113 involved a residue that was located in close proximity to the residues Gly155 and Ala157, which were previously found to be associated with linezolid resistance. Substitutions at these positions were reported to cause resistance by abolishing linezolid binding to its target [23], [24]. Thus, the presence of the *cfr* gene and L3 substitutions in both *S. haemolyticus* 09D279 and *S. cohnii* NDM113 may act synergistically.

### Antimicrobial resistance patterns, PFGE analysis, and plasmid analysis

All four *cfr*-carrying isolates exhibited resistance to linezolid, chloramphenicol, and clindamycin, and showed elevated MICs to florfenicol, tiamulin, quinupristin/dalfopristin and virginiamycin M1, all of which are consistent with the resistance phenotype caused by the *cfr* gene. Additionally, these isolates were resistant to oxacillin, cefoxitin, and levofloxacin, but susceptible to rifampicin, tigecycline, teicoplanin, and vancomycin (Table 1). All four oxacillin-resistant isolates harbored the *mecA* gene and were therefore considered to be methicillin-resistant coagulase-negative staphylococci (MRCoNS). The MIC results showed that the three *S. cohnii* isolates exhibited resistance to erythromycin and susceptibility to sulfamethoxazole-trimethoprim, while the *S. haemolyticus* 09D279 isolate was susceptible to erythromycin but resistant to trimethoprim-sulfamethoxazole (Table 1). The PFGE results suggested that the three *S. cohnii* isolates represented two different clones, with isolates 09D253 and 09D363 recovered from different patients in hospital A belonging to the same clone (data not shown).

**Table 1:** Antimicrobial susceptibility profiles of linezolid-resistant clinical strains.

Staphylococcal isolates					MIC (in µg/ml)*												
	LZD	CHL	CLI	FFC	TIA	VM1	Q-D	OXA	FOX	LEV	ERY	RIF	TGC	SXT	GEN	VAN	TEC
*S. haemolyticus* 09D279	8	256	32	256	256	32	2	≥512	256	16	0.25	0.008	0.125	64	8	0.5	4
*S. cohnii* 09D253	32	128	≥512	256	128	>128	4	1	8	8	≥512	0.016	0.125	0.125	1	0.5	2
*S. cohnii* 09D363	32	128	≥512	256	128	128	4	0.5	8	8	≥512	0.008	0.125	0.125	2	0.5	2
*S. cohnii* NDM113	32	128	256	256	128	128	8	128	32	8	256	0.008	0.25	0.125	32	0.5	1
*S. aureus* RN4220	2	8	0.125	1	0.5	0.5	0.25	0.125	1	0.25	0.125	0.008	0.125	0.125	0.125	2	0.5
*S. aureus* RN4220 + pSS-02-like (09D279)	8	>256	>256	128	64	4	2	0.125	1	0.25	0.125	0.008	0.125	0.125	0.125	2	0.5
*S. aureus* RN4220 + pSS-02-like (09D253)	8	>256	>256	128	64	4	2	0.125	1	0.25	0.125	0.008	0.125	0.125	0.125	2	0.5
*S. aureus* RN4220 + pSS-02-like (09D363)	8	>256	>256	128	64	4	1	0.125	1	0.25	0.25	0.008	0.125	0.125	0.125	2	0.5
*S. aureus* RN4220 + pSS-02-like (NDM113)	8	>256	>256	128	64	4	2	0.125	1	0.25	0.125	0.008	0.125	0.125	0.25	2	0.5
*S. aureus* RN4220 + pSS-02	8	>256	>256	256	64	4	2	0.12	1	0.25	0.125	0.016	0.125	0.125	0.25	2	0.5

* LZD, linezolid; CHL, chloramphenicol, CLI, clindamycin; FFC, florfenicol; TIA, tiamulin; VM1, virginiamycin M1; Q-D, quinupristin/dalfopristin; OXA, oxacillin; FOX, cefoxitin; LEV, levofloxacin; ERY, erythromycin; RIF, rifampicin; TGC, tigecycline; SXT, sulfamethoxazole-trimethoprim; GEN, gentamicin; VAN, vancomycin; TEC, teicoplanin.

**Figure 1:**
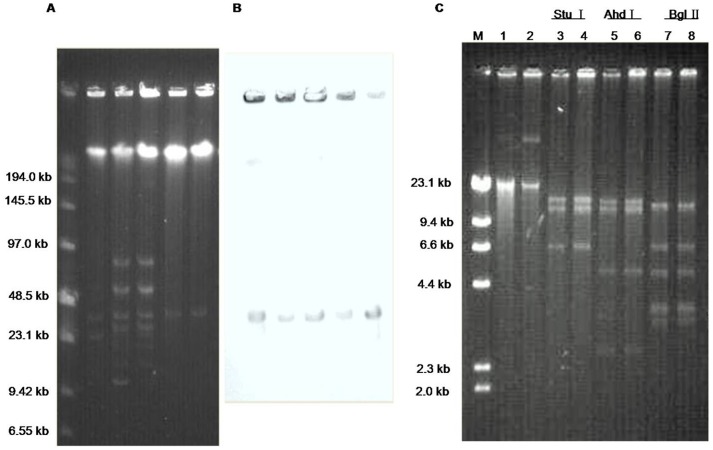
Identification and characterization of the pSS-02-like *cfr*-carrying plasmid found in the four MRCoNS isolates in comparison to the original plasmid pSS-02. (A) S1 nuclease-PFGE (B) Southern blot hybridization with the *cfr* probe. Lane 1:*S. haemolyticus* 09D279, Lane 2: *S. cohnii* 09D253, Lane 3: *S. cohnii* 09D363; Lane 4: *S. cohnii* NDM113; Lane 5: RN4220 + pSS-02. (C) Restriction digests of the pSS-02-like plasmid extracted from transformants originating from *S. haemolyticus* 09D279 (lanes 1, 3, 5, and 7) and the original plasmid pSS-02 extracted from transformants originating from *S. saprophyticus* 2-87 (lanes 2, 4, 6, and 8). The restriction enzymes used to digest the plasmids are indicated above the respective lanes.

Although the *cfr* gene can also be located in chromosomal DNA, it is mostly plasmid-borne [25]. S1 nuclease-PFGE analysis revealed that each of the four isolates harbored multiple plasmids of different sizes. The two clonally related *S. cohnii* isolates displayed the similar plasmid profile (Figure 1A). In addition, we found that the *cfr* probe hybridized to a plasmid of ca. 35.4 kb in all four clinical isolates (Figure 1B). Electrotransformation into *S. aureus* RN4220 was successful and transformants carrying only the 35.4-kb plasmid were obtained from each of the four MRCoNS isolates. A similar-sized plasmid, designated pSS-02, has previously been identified in *S. sciuri* and *S. saprophyticus* isolates of porcine origin [19].

MIC determination of these transformants identified the same resistance pattern, namely resistance to linezolid, chloramphenicol and clindamycin, and presented elevated MICs of florfenicol, tiamulin, quinupristin/dalfopristin and virginiamycin M1 in all transformants (Table 1). This resistance phenotype is indicative for the presence and the functional activity of the *cfr* gene. In addition, the transformants that carried these *cfr* plasmids had identical MIC values when compared with the previously described RN4220 transformants harboring plasmid pSS-02 (Table 1). Moreover, indistinguishable AhdI, BglII, EcoRI, StuI and XhoI restriction patterns were observed in both pSS-02 and the *cfr*-carrying plasmids extracted from transformants in this study. Some of the restriction patterns are shown in Figure 1C. Taken together, the aforementioned observations strongly suggest that a plasmid which is at least very closely related to plasmid pSS-02 of porcine origin, was also present in these four clinical MRCoNS isolates from China.

### Genetic environment of the *cfr* gene on plasmid pSS-02

**Figure 2:**
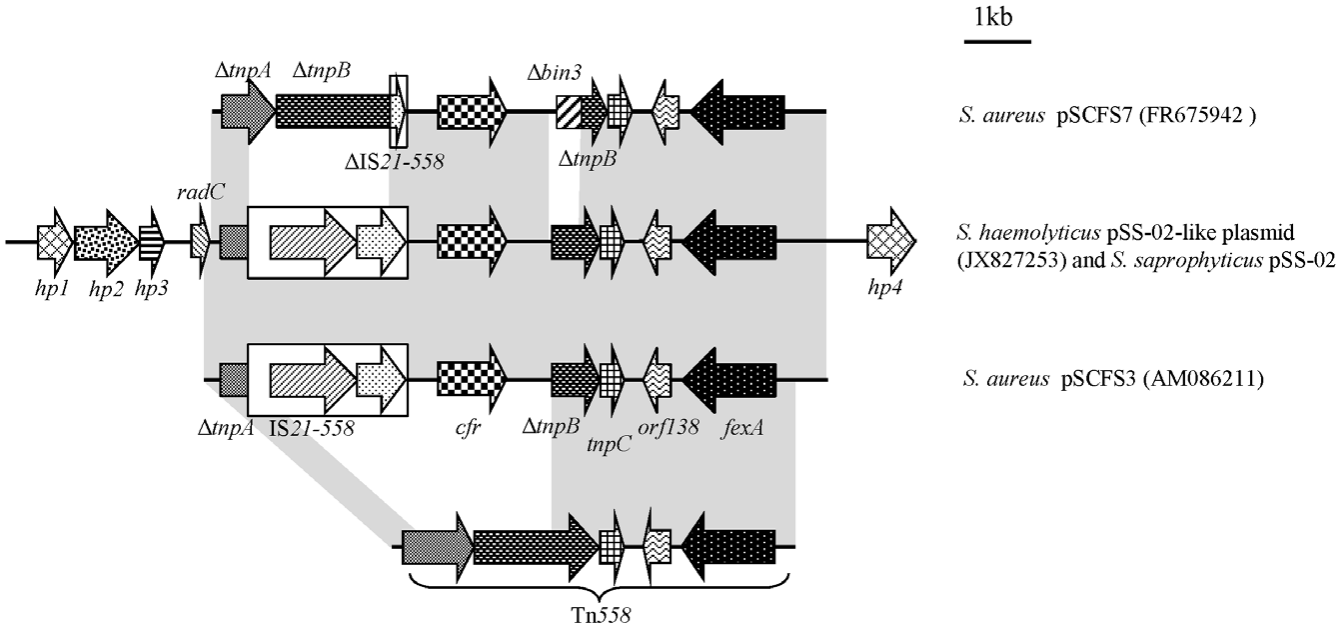
Schematic representation of the genetic environment of the *cfr* gene in pSS-02-like, pSS-02, pSCFS3 and pSCFS7 plasmids. Arrows indicate the positions and directions of gene transcription. Regions exhibiting >99% homology are marked with gray shading. Δ indicates a truncated gene. The distance scale (in kilobases) is displayed in the upper right-hand corner.

To gain insight into the genetic environment of the *cfr* gene on the pSS-02-like plasmid extracted from a *S. haemolyticus* 09D279 transformant (patient origin), a 13,976 bp segment encompassing the *cfr* gene was sequenced. Of plasmid pSS-02 from porcine *S. saprophyticus*, a 8.5-kb segment including the *cfr* gene had been sequenced. For a better comparison, sequence analysis of the original plasmid pSS-02 had been extended to match the sequence of the pSS-02-like plasmid from human *S. haemolyticus* 09D279. A comparison of these two almost 14-kb fragments revealed 100% nucleotide sequence identity (Figure 2). Subsequently, this segment was sequenced from plasmids of the remaining three clinical *S. cohnii* isolates and shown to be also identical to the aforementioned two sequences. The detection of identical *cfr*-carrying segments on plasmids isolated from CoNS of porcine and MRCoNS of human origin provided further confirmation that plasmid pSS-02 or a closely related derivative is exchanged between animal and human staphylococci.

In this 14-kb segment from pSS-02, a 9.5-kb region containing an IS*21-558* insertion sequence and the *cfr* gene, were integrated into a Tn*558* element, thereby truncating the transposase genes *tnpA* and *tnpB*. This 9.5-kb region resembled closely (99.8% identity; 9487/9503 bp) the corresponding region of the ca. 34.7-kb pSCFS3 (GenBank accession number AM086211), which originated from bovine and porcine *Staphylococcus lentus* and porcine *S. aureus* (including one MRSA ST398) from Germany; while differed slightly from the corresponding region of the ca. 45-kb plasmid pSCFS7, which originated from a linezolid-resistant ST8-MRSA-IVa/USA300 recovered from a skin scalp abscess of Irish male (Figure 2) [10], [26]. Moreover, we previously reported that pSS-02 might be similar to pSCFS3 based on their similar plasmid sizes, BglII digest fragment patterns, and the absence of other antimicrobial resistance genes [19]. Immediately upstream of the truncated *tnpA* sequence in pSS-02, we found the *radC* gene that encoded an 109 amino acid DNA repair protein that shared 94.5% identity with the 114 amino acid RadC protein from the *S. aureus* plasmid pSK73 (GenBank accession number GQ915269). Three open reading frames encoding hypothetical proteins were identified upstream of the *radC* gene and another downstream of the *fexA* gene on Tn*558*. The observation that similar plasmids (pSS-02 and pSCFS3) were found in porcine isolates of *S. sciuri* and *S. saprophyticus*, and clinical samples of *S. cohnii* and *S. haemolyticus* in China, as well as bovine and porcine *S. lentus* and porcine *S. aureus* in Europe, provides further confirmation of how widely these plasmids are disseminated.

In conclusion, this is the first report of four clinical linezolid-resistant MRCoNS in which a *cfr*-carrying plasmid previously found in staphylococci from food producing animal was detected. This finding has important implications as it showed that closely related – if not identical – plasmids can be exchanged between CoNS from animals and MRCoNS from humans and that these MRCoNS can be involved in severe infections in humans. When and under which conditions this plasmid transfer has occurred remains to be answered. An additional concern is that *S. cohnii* and *S. haemolyticus* isolates that harbor this *cfr*-carrying plasmid could act as reservoirs for the *cfr* gene in nosocomial environments. Although a very low prevalence (0.55%, 4/713) of the *cfr* gene was observed in the clinical staphylococcal isolates from the Mohnarin study 2009–2010, continued surveillance of the dissemination of the *cfr* gene in Gram-positive bacteria from hospital patients is urgently needed in China.
